# Iron-Restricted Diet Affects Brain Ferritin Levels, Dopamine Metabolism and Cellular Prion Protein in a Region-Specific Manner

**DOI:** 10.3389/fnmol.2017.00145

**Published:** 2017-05-17

**Authors:** Jessica M. V. Pino, Marcio H. M. da Luz, Hanna K. M. Antunes, Sara Q. de Campos Giampá, Vilma R. Martins, Kil S. Lee

**Affiliations:** ^1^Departamento de Bioquímica, Universidade Federal de São PauloSão Paulo, Brazil; ^2^Departamento de Psicobiologia, Universidade Federal de São PauloSão Paulo, Brazil; ^3^Departamento de Biociências, Universidade Federal de São PauloSão Paulo, Brazil; ^4^AC Camargo Cancer CenterSão Paulo, Brazil

**Keywords:** iron deficiency, dopamine, ferritin, prion protein, α-synuclein

## Abstract

Iron is an essential micronutrient for several physiological functions, including the regulation of dopaminergic neurotransmission. On the other hand, both iron, and dopamine can affect the folding and aggregation of proteins related with neurodegenerative diseases, such as cellular prion protein (PrP^C^) and α-synuclein, suggesting that deregulation of iron homeostasis and the consequential disturbance of dopamine metabolism can be a risk factor for conformational diseases. These proteins, in turn, are known to participate in the regulation of iron and dopamine metabolism. In this study, we evaluated the effects of dietary iron restriction on brain ferritin levels, dopamine metabolism, and the expression levels of PrP^C^ and α-synuclein. To achieve this goal, C57BL/6 mice were fed with iron restricted diet (IR) or with normal diet (CTL) for 1 month. IR reduced iron and ferritin levels in liver. Ferritin reduction was also observed in the hippocampus. However, in the striatum of IR group, ferritin level was increased, suggesting that under iron-deficient condition, each brain area might acquire distinct capacity to store iron. Increased lipid peroxidation was observed only in hippocampus of IR group, where ferritin level was reduced. IR also generated discrete results regarding dopamine metabolism of distinct brain regions: in striatum, the level of dopamine metabolites (DOPAC and HVA) was reduced; in prefrontal cortex, only HVA was increased along with the enhanced MAO-A activity; in hippocampus, no alterations were observed. PrP^C^ levels were increased only in the striatum of IR group, where ferritin level was also increased. PrP^C^ is known to play roles in iron uptake. Thus, the increase of PrP^C^ in striatum of IR group might be related to the increased ferritin level. α-synuclein was not altered in any regions. Abnormal accumulation of ferritin, increased MAO-A activity or lipid peroxidation are molecular features observed in several neurological disorders. Our findings show that nutritional iron deficiency produces these molecular alterations in a region-specific manner and provide new insight into the variety of molecular pathways that can lead to distinct neurological symptoms upon iron deficiency. Thus, adequate iron supplementation is essential for brain health and prevention of neurological diseases.

## Introduction

Iron is an important micronutrient that participates in several vital cellular functions such as oxygen transport and cellular respiration. Moreover, iron is a cofactor for numerous enzymes. Despite its importance, free iron is known to cause oxidative damages in cellular environment due to its ability to generate free radicals through Fenton reaction (Zecca et al., 2004; Belaidi and Bush, 2015). Indeed, abnormal accumulation of iron in brain has been pointed out as one of the factors that accelerate the aggregation of proteins associated with neurodegenerative diseases, such as cellular prion protein (PrP^C^) and α-synuclein (Golts et al., 2002; Das et al., 2010). Moreover, iron can bind to protein aggregates and enhance their toxicity by attributing redox activity to these aggregates (Liu et al., 2011).

On the other hand, iron deficiency can turn out the aerobic respiration inefficient leading to reduced energy production and accumulation of reactive oxygen species, which can culminate into mitochondrial dysfunction (Masini et al., 1994; Walter et al., 2002). Iron deficiency can also cause deregulation of monoaminergic system. Inhibition of iron uptake into dopaminergic neurons not only caused mitochondrial damage, but also reduced dopamine levels and evoked abnormal activity of dopamine receptors (Matak et al., 2016). Several studies have also reported that nutritional iron deficiency alters dopaminergic neurotransmission, increasing the concentration of extracellular dopamine and reducing the activity of dopamine transporter (DAT) and D2 receptor in striatum (Youdim et al., 1989; Beard et al., 1994; Bianco et al., 2008; Unger et al., 2014). Reduced DAT expression has been also reported in the ventral midbrain (Bianco et al., 2008). These alterations appear to contribute to the memory impairment, attention deficiency and learning problems frequently observed in iron-deficient population (Youdim et al., 1989; Asanuma et al., 2003; Radlowski and Johnson, 2013; Gupta, 2016; Scott and Murray-Kolb, 2016). However, how nutritional iron deficiency affects the brain iron metabolism, particularly in dopaminergic regions, is poorly understood. Brain appears to have a greater tendency to retain iron and resist to nutritional iron deficiency due to the slow exchange of iron out of brain (Youdim et al., 1989; Bradbury, 1997). However, a few studies have demonstrated that dietary iron restriction reduces the iron levels of ventral midbrain and striatum in young rats (Bianco et al., 2008; Unger et al., 2014), while others claimed that iron levels of striatum were not altered after similar treatment (Erikson et al., 1997). Thus, the effects of dietary iron on the iron metabolism in dopaminergic neurons need to be better investigated.

Dopamine is a neurotransmitter that participates in the regulation of motor coordination, responses to rewarding experiences, attention and mood (Russo and Nestler, 2013; Bissonette and Roesch, 2016). At the same time, dopamine can be neurotoxic, being readily oxidized by non-enzymatic reactions, which generate reactive molecules, such as dopamine quinone (Lotharius and Brundin, 2002; Asanuma et al., 2003). Oxidative dopamine metabolites are known to promote the aggregation of α-synuclein into non-ordered toxic oligomers, and these molecular alterations are closely associated with pathogenesis of Parkinson's disease (Lotharius and Brundin, 2002; Yamakawa et al., 2010). Dopamine can also induce the aggregation of PrP^C^, another aggregation-prone protein associated with transmissible spongiform encephalopathies, a fatal neurodegenerative disease (da Luz et al., 2015).

Altogether, these previous findings suggest that the aggregation of α-synuclein and PrP^C^ can be promoted either by iron or by dopamine, and both excess and deficiency of iron can be a risk factor for conformational diseases through distinct molecular mechanisms. It is possible that the distinct subcellular localization of the proteins and the site of iron and/or dopamine accumulation contribute to the specificity of pathogenic pathways. Thus, in this study, we evaluated how dietary iron restriction influences brain ferritin (a main iron storage protein) and dopamine metabolism in striatum, hippocampus and prefrontal cortex, three regions that receive afferents from midbrain dopaminergic neurons (Yetnikoff et al., 2014) and investigated how altered iron and dopamine metabolism can affect the expression level and solubility of PrP^C^, a protein that is anchored to the outer leaflet of the plasma membrane. As mentioned above, dietary iron restriction is known to increase the concentration of extracellular dopamine. Since the accumulation of α-synuclein occur due to the intracellular dopamine, in order to check the specificity of the iron restriction effects on proteins of distinct subcellular comportments, we also analyzed the level of α-synuclein, which is a cytosolic protein.

## Materials and methods

### Dietary iron restriction

Three-month-old male C57BL/6 mice were used in this study. The animals were randomly distributed into two groups: control group (CTL) that was fed with normal diet containing 45 ppm of iron (Research Diets, USA, cat # D100012G) and IR group that was fed with iron restricted diet containing approximately 3 ppm of iron (Research Diets, USA, cat # D0372501N) for 4 weeks. This protocol was chosen based on the previous studies (Youdim et al., 1989; Beard et al., 1994; Bianco et al., 2008; Kamei et al., 2013; Unger et al., 2014). During the experimental period, the animals were housed in a temperature-controlled room at 23 ± 2°C with a 12/12-h light/dark cycle, and water and food were supplied *ad libitum*. The food was removed 2 h before euthanasia. All experimental procedures were approved by the Research Ethics Committee of UNIFESP (CEUA N 9806251113). All experimental procedures complied with the Guide for the Care and Use of Laboratory Animals (National Research Council, NIH Publication No. 85-23, 2011 revision). This study does not contain any individual persons data. The datasets used and/or analyzed during the current study available from the corresponding author on reasonable request.

### Iron status

After blood clotting, samples were centrifuged at 1,100 × g for 10 min and serum was collected. For liver, small peripheral piece of the organ was dissected to avoid harvesting main veins and the piece were extensively rinsed in PBS prior to lysis. Liver, striatum, prefrontal cortex and hippocampus were homogenized in phosphate buffered saline (PBS). Total amount of iron in serum and in liver was measured using Ferene S provided in the commercially available colorimetric assay kit (BioVision, USA, cat# K390-100). Buffers provided in this kit dissociate iron from iron carrier proteins, and after reduction of ferric iron, total iron is detected as ferrous form. Ferritin and transferrin receptor were quantified using enzyme linked immunosorbent assay kits: Ferritin ELISA kit (Abnova, USA, cat # KA1941) and Transferrin receptor ELISA kit (Mybiosource, USA, cat # MBS026588). All experiments were performed according to the instructions of the manufacturers.

### Lipid peroxidation

Striatum, prefrontal cortex and hippocampus were homogenized in RIPA buffer (Tris 50 mM pH 8, NaCl 150 mM, SDS 1%, triton-x100 1%, Sodium deoxycholate 0.5%). After the centrifugation of homogenates at 1,600 × g for 10 min, post-nuclear supernatant was collected and the concentration of malondialdehyde was determined using fluorimetric TBARS assay kit (Cayman, USA, cat # 10009055).

### Dopamine and its metabolites

Levels of dopamine and its metabolites were determined using HPLC-ECD system composed of ECD-700 detector, ECD-700 pump and ATC-700 temperature controller (Eicom, Japan). Striatum, prefrontal cortex and hippocampus were homogenized in 0.2 M HClO_4_ and centrifuged at 20,000 × g for 15 min. Supernatant pH was adjusted to 3 using sodium acetate 2 M, and then filtered through 0.22 μm PVDF membrane. Samples (10 μl) were injected every 25 min onto SC-3ODS HPLC column coupled to pre-column packed with AC-ODS, using the auto sampler 700 (Eicom, Japan). The isocratic mobile phase consisted of citric acid 42 mM, sodium acetate 38 mM, EDTA-Na_2_ 13 μM and sodium octanesulfonate 1 mM. The flow rate was 340 μl/min. The analytes were detected using graphite working electrode WE-3G set at +750 mV vs. Ag/AgCl reference electrode.

### Monoamine oxidase (MAO) activity

Striatum and prefrontal cortex were homogenized in 50 μl of MAO buffer provided in Monoamine Oxidase Activity Fluorometric Assay Kit (BioVision, USA, Cat# K795-100) and supernatant was recovered after the centrifugation at 1,000 × g for 10 min. After combining samples, substrate and fluorescent probe for H_2_O_2_ provided in the kit, the fluorescence was measured every 1 min during an hour. MAO-A activity was determined using selective MAO-B inhibitor selegilin, also provided in the kit.

### Western blot

Tissues were homogenized in lysis buffer [Tris 50 mM pH 8, NaCl 150 mM, EDTA 10 mM, triton-x100 1%, Sodium deoxycholate 0.5%, cOmplete™ Protease Inhibitor Cocktail (Roche, Brazil), Phosphatase Inhibitor Cocktail set II (Millipore, Brazil)] and post-nuclear supernatant was collected after the centrifugation at 3,000 × g for 5 min. Following SDS-PAGE, proteins were transferred to PVDF membrane and the protein of interest was detected using primary antibodies: SAF32 for PrP^C^ (Cayman Chemical, USA, Cat #189720); anti- α-synuclein (Cell Signaling Technology, USA, Cat # 2642); anti- tyrosine hydroxylase (TH) (Cell Signaling Technology, USA, Cat # 27925); anti- DAT (EDM Millipore, USA, Cat # MAB369); anti- GAPDH (Cell Signaling Technology, USA, Cat #2118). The signals were revealed with HRP-conjugated secondary antibodies and Luminata™ Forte Western HRP Substrate (Millipore, USA, Cat#WBLUF0500). Digital images of membranes were acquired using Alliance Mini gel documentation system (UVITEC, UK). Band intensity was quantified using software UVIband (UVITEC, UK). The intensity of the protein of interest was normalized with the GAPDH band and data were presented as percentage of mean of control group.

### Ultracentrifugation

Post-nuclear supernatant prepared in PBS were incubated with 1% sarkosyl for 10 min on ice and centrifuged at 30,000 r.p.m. for 3 h at 4°C using Optima L-100 Ultracentrifuge and Sw55TI rotor (Beckman Coulter, Inc, USA). After collecting soluble fractions (supernatant), insoluble fractions (pellets) were rinsed with PBS and dissolved in 4x sample buffer (8% SDS, Tris HCl 250 mM, pH 6.8, 40% Glycerol, 0.08 mg/ml bromophenol blue and 1.4 M β-mercaptoetanol) with vigorous vortexing and sonication. One twentieth of the supernatant volume was used to dissolve the pellets.

### Data analysis

For each experiment, 3–10 independent biological samples per group were analyzed depending on the availability of samples. All individual observations were presented in scatter plots. Data were analyzed using non-parametric Mann-Whitney *U*-tests and groups were considered significantly different when the p value was lower than 0.05. All statistical analyses presented in this study were performed including potential outliers. However, the exclusion of the potential outliers did not affect the significance level of the results (data not shown).

## Results

### Body weight and iron status

Animals fed with iron-depleted chow (IR) for 30 days gained 2.5 ± 0.9 g (mean ± SD, *n* = 10) throughout the experimental period. This weight gain was not significantly different from that observed in control group (CTL): 3.0 ± 1.3 g (mean ± SD, *n* = 10). These results were replicated in further independent experiments.

**Figure 1:**
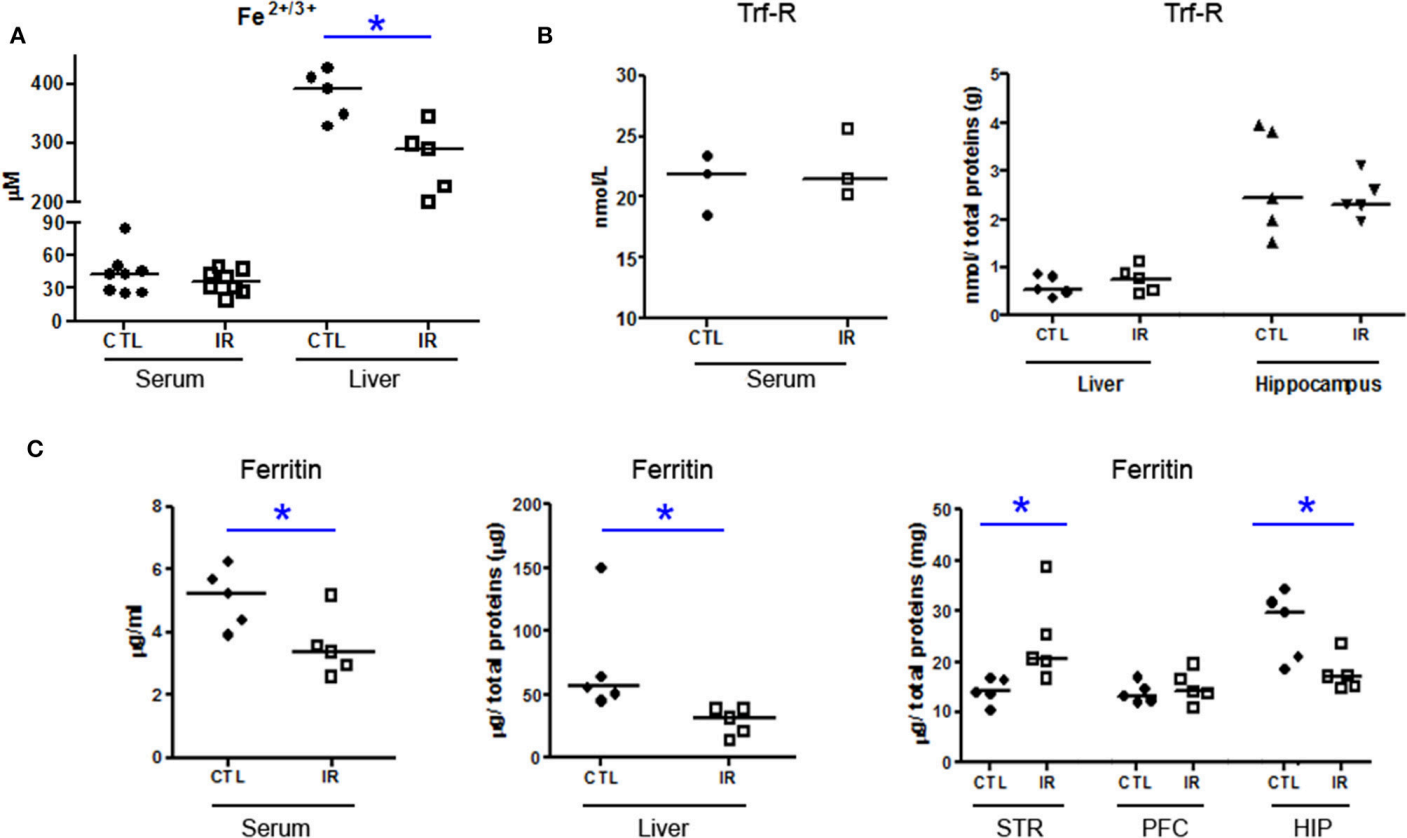
**Iron Metabolism. (A)** Total amount of Fe^2+/3+^ in serum and liver of CTL and IR animals was measured using the colorimetric method (serum *n* = 8 per group; liver *n* = 5 per group). **(B,C)** Levels of transferrin receptor (Trf-R) and ferritin were determined by ELISA. (serum Trf-R *n* = 3 per group; liver and hippocampus Trf-R *n* = 5 per group; serum ferritin *n* = 5 per group; liver, striatum (STR), prefrontal cortex (PFC), and hippocampus (HIP) ferritin *n* = 5 per group). Each dot represents result of an animal and horizontal line represents median of the group. ^*^p < 0.05.

IR group presented reduced iron levels in liver (Figure 1A), and reduced ferritin in serum and liver (Figure 1C) compared to CTL group, but the concentration of serum iron and transferrin receptor were not altered (Figures 1A,B). These data suggest that 30 days of dietary iron restriction caused mild iron deficiency.

Ferritin level was also reduced in the hippocampus of IR group compared to CTL, but in the striatum, ferritin level was increased with dietary iron restriction (Figure 1C). In prefrontal cortex, ferritin level was not altered (Figure 1C). Although we were not able to detect iron in brain tissues using the colorimetric assay, ferritin levels of each region indirectly indicate that the depletion of the brain iron pool is controlled in a region-specific manner.

**Figure 2:**
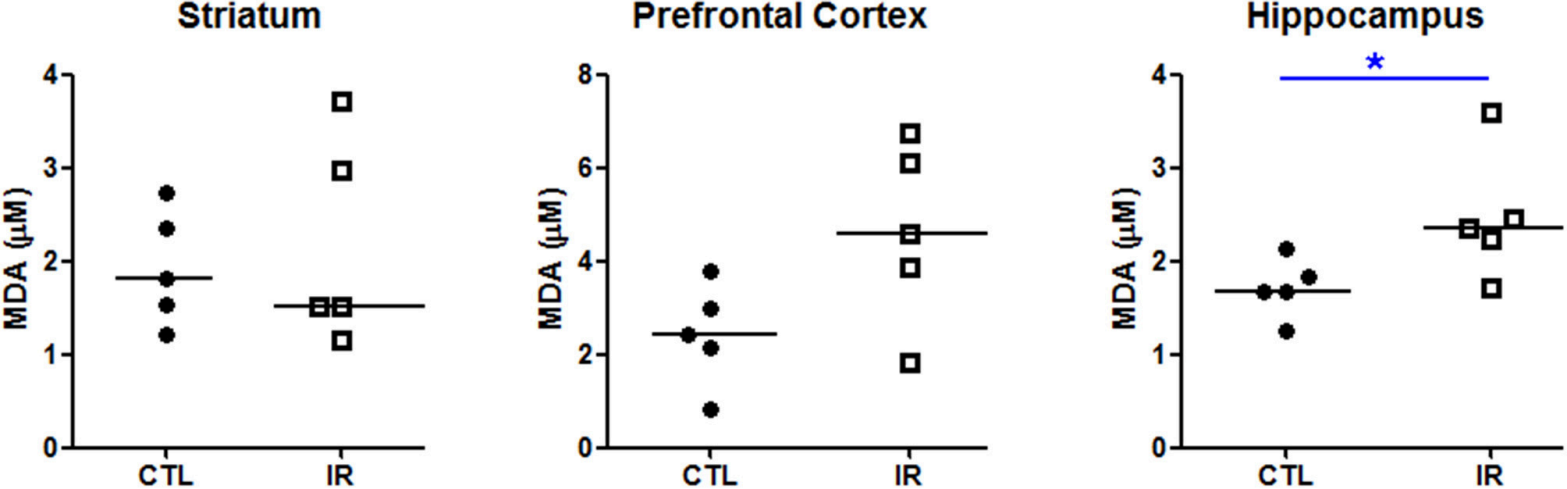
**Lipid peroxidation**. The concentration of malondialdehyde (MDA), a product of lipid peroxidation, was measured in striatum, prefrontal cortex and hippocampus using thiobarbituric acid (*n* = 5 per group). Each dot represents result of an animal and horizontal line represents median of the group. ^*^p < 0.05.

To verify the effects of this heterogeneous iron depletion on redox balance, we evaluated the extent of lipid peroxidation in each region using thiobarbituric acid. As shown in Figure 2, only hippocampus of IR group, which had lower levels of ferritin, presented higher degree of lipid peroxidation compared to CTL group (Figure 2). Significant alterations were not observed in other regions.

### Effects of dietary iron restriction on dopamine metabolism

Effects of iron restriction on dopamine metabolism have been extensively investigated in human and in animal models, especially in the nigrostriatal pathway (Youdim et al., 1989; Bianco et al., 2008; Unger et al., 2014). To confirm and complement these previous observations, we measured total dopamine levels in three brain areas.

**Figure 3:**
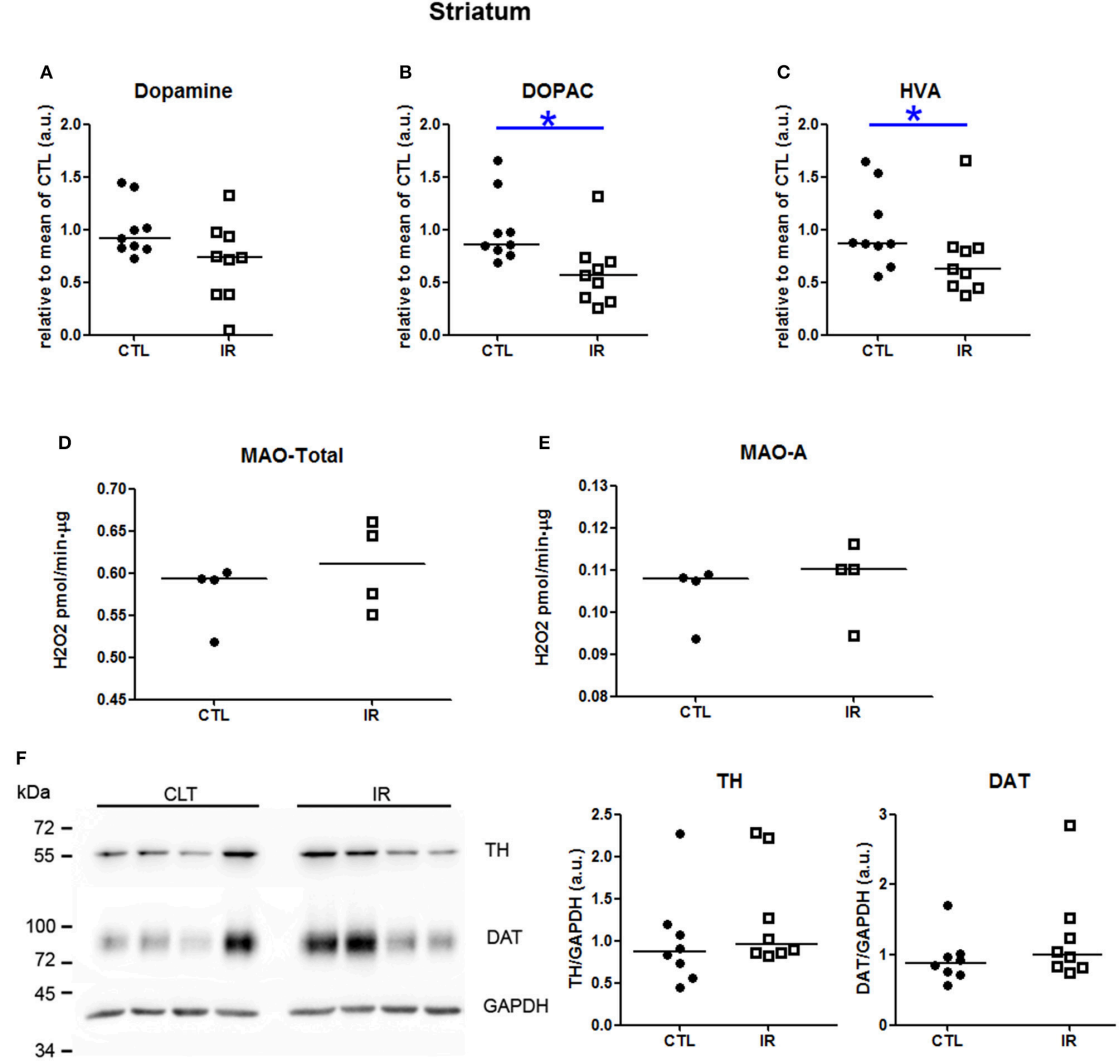
**Metabolism of dopamine in striatum**. **(A–C)** Dopamine, DOPAC and HVA levels were measured in two independent experiments. In each experiment, 4–5 animals were used for each group, and the concentration of each sample was normalized by mean of CTL of respective experiment. The scatter plots show combination of two experiments (*n* = 9 per group). **(D)** Total MAO activity was assessed by monitoring the variation of the concentration of H_2_O_2_, which is a byproduct of the reaction (*n* = 4 per group). **(E)** MAO-A activity was measured using selegilin, a MAO-B selective inhibitor (*n* = 4 per group). **(F)** TH and DAT levels were evaluated by Western blotting. Left panel shows a representative image. The intensity of TH and DAT bands was normalized by the intensity of GAPDH bands (*n* = 8 per group). Each dot represents result of an animal and horizontal line represents median of the group. ^*^p < 0.05.

In striatum, dopamine levels were not altered by iron restricted diet (Figure 3A). However, the levels of 3,4-Dihydroxyphenylacetic acid (DOPAC) and homovanillic acid (HVA) were reduced in IR group compared to CTL group (Figures 3B,C). This reduction was neither due to the altered activity of monoamine oxidase (MAO) (Figures 3D,E) nor due to the expression level of tyrosine hydroxylase (TH) (Figure 3F). Iron restriction also did not alter the level of DAT (Figure 3F). These results were somewhat expected based on the previous findings, except DAT expression which was expected to be lower in IR group (Bianco et al., 2008; Unger et al., 2014). This subtle difference might have occurred due to the distinct age and species of animals used in each study. In previous studies, dorsal striatum of 65 days old rats was used, while in our study, whole striatum of 4 month old mice was used. Similarity of the results observed in two distinct animal models indicates that the reduction of dopamine metabolites in iron deficient animals is a robust phenomenon.

**Figure 4:**
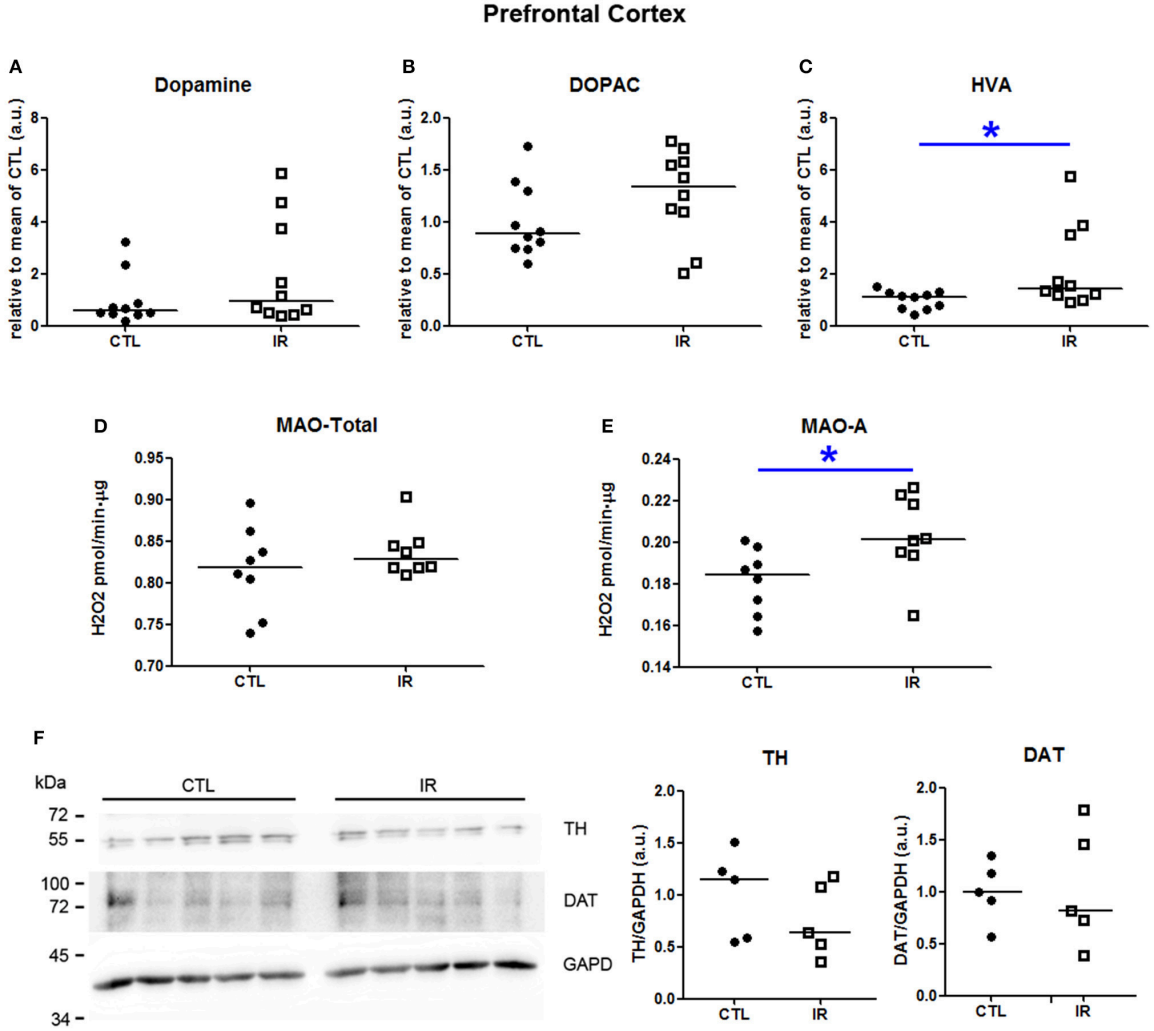
**Metabolism of dopamine in prefrontal cortex**. (**A–C)** Dopamine, DOPAC and HVA levels were measured in two independent experiments. In each experiment, five animals were used for each group, and the concentration of each sample was normalized by mean of CTL of respective experiment. The scatter plots show combination of two experiments (*n* = 10 per group). **(D)** Total MAO activity was assessed by monitoring the variation of the concentration of H_2_O_2_, which is a byproduct of the reaction (*n* = 8 per group). **(E)** MAO-A activity was measured using selegilin, a MAO-B selective inhibitor (*n* = 8 per group). **(F)** TH and DAT levels were evaluated by Western blotting. Left panel shows a representative image. The intensity of TH and DAT bands was normalized by the intensity of GAPDH bands (*n* = 5 per group). Each dot represents result of an animal and horizontal line represents median of the group. ^*^p < 0.05.

In prefrontal cortex, iron restriction promoted an increase of HVA levels in IR group compared to CTL group (Figure 4C). Dopamine and DOPAC levels did not differ significantly between the groups (Figures 4A,B). The increase of HVA might be caused due to the increased MAO-A activity (Figure 4E). Total MAO activity was not altered by iron restriction (Figure 4D). Neither TH nor DAT were affected (Figure 4F).

**Figure 5:**
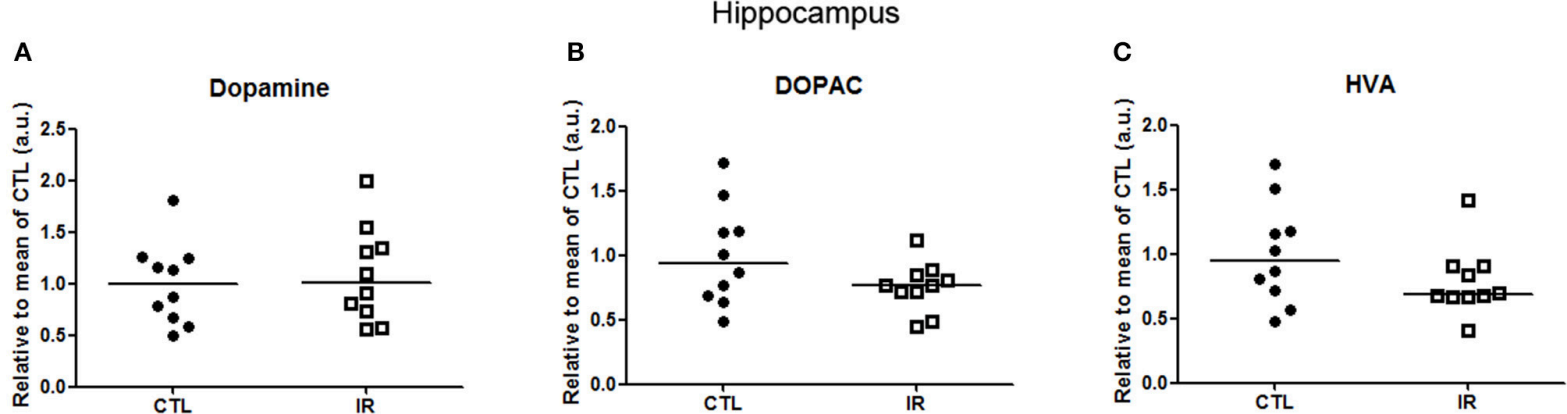
**Metabolism of dopamine in hippocampus**. **(A–C)** Dopamine, DOPAC and HVA levels were measured in two independent experiments. In each experiment, 5 animals were used for each group, and the concentration of each sample was normalized by mean of CTL of respective experiment. The scatter plots show combination of two experiments (*n* = 10 per group).

In hippocampus, levels of dopamine and its metabolites were not altered with iron restricted diet (Figure 5). Moreover, we were not able to detect DAT or TH in a reliable manner. Hippocampus receives only a small portion of dopaminergic innervation from ventral tegmental area (VTA) (Gasbarri et al., 1994). Thus, it is expected that the detection of TH or DAT in hippocampus would be more difficult than in other regions.

### Effects of dietary iron restriction on PrP^C^ and α-synuclein

Since both PrP^C^ and α-synuclein play roles in iron and dopamine metabolism (Wong and Duce, 2014; Haldar et al., 2015; da Luz et al., 2015, 2016; Benskey et al., 2016), we investigated how dietary iron restriction affected the expression level of these proteins in each region by Western Blots. For the quantification of PrP^C^, we measured the intensity of three bands that correspond to di-, mono-, and unglycosylated forms. The band intensity of protein of interest was normalized by GAPDH given that the alteration of GAPDH expression in IR group was not noticeable. To enhance the reliability of the results, each independent biological sample was assayed at least in duplicate and the mean of replicates was used for statistical analysis and presented in the graphs.

**Figure 6:**
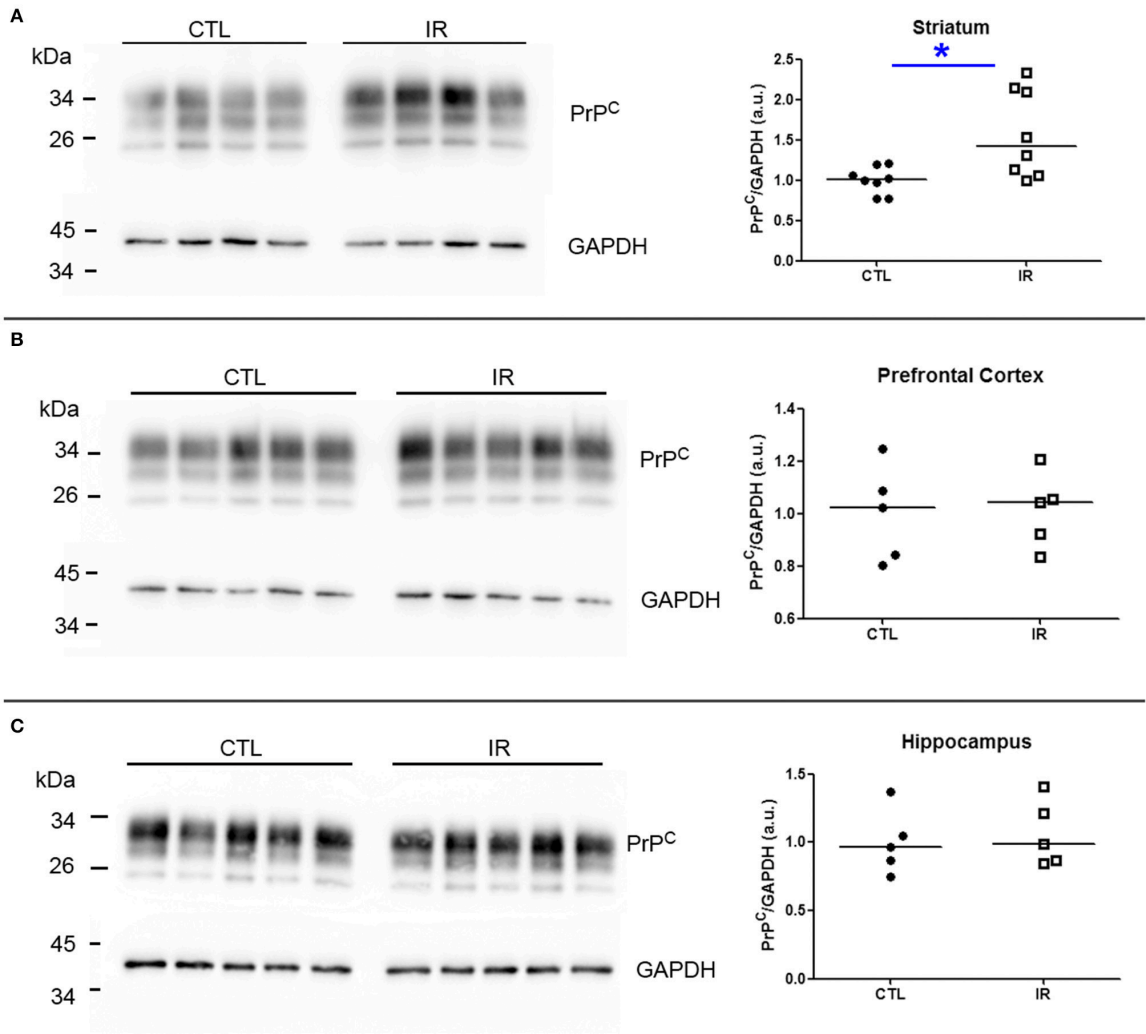
**Analysis of PrP^C^ levels**. PrP^C^ levels were analyzed by Western blotting. Left panels show representative images of each region. The intensity of PrP^C^ bands was normalized by the intensity of respective GAPDH band. Each dot represents result of an animal and horizontal line represents median of the group. **(A)** Striatum (*n* = 8 per group). **(B)** Prefrontal cortex (*n* = 5 per group). **(C)** Hippocampus (*n* = 5 per group). ^*^p < 0.05.

**Figure 7:**
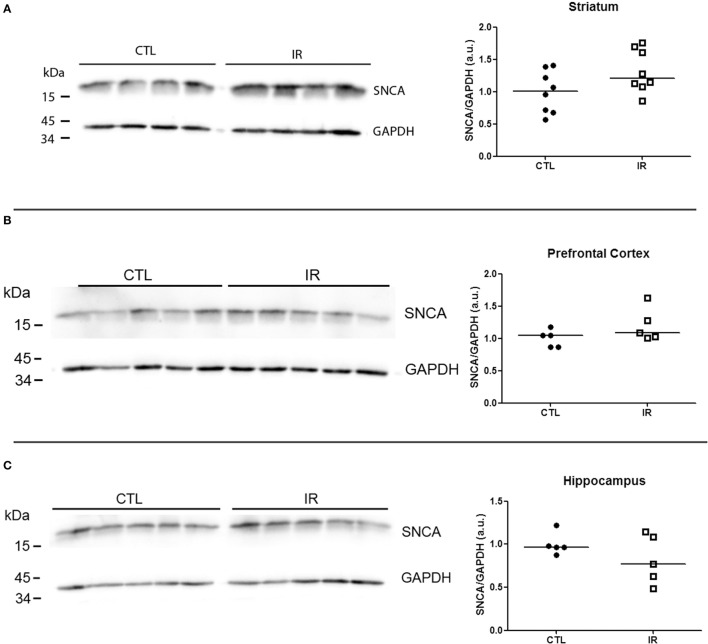
**Analysis of α-synuclein levels**. Levels of α-synuclein were analyzed by Western blotting. Left panels show representative images of each region. The intensity of α-synuclein bands was normalized by the intensity of respective GAPDH band. Each dot represents result of an animal and horizontal line represents median of the group. **(A)** Striatum (*n* = 8 per group). **(B)** Prefrontal cortex (*n* = 5 per group). **(C)** Hippocampus (*n* = 5 per group).

In striatum, PrP^C^ levels were increased in IR group compared to CTL group (Figure 6A). In other regions, iron restriction did not produce significant effects on PrP^C^ levels (Figures 6B,C). With regard to α-synuclein, none of the regions presented significant alteration in IR group (Figures 7A–C).

**Figure 8:**
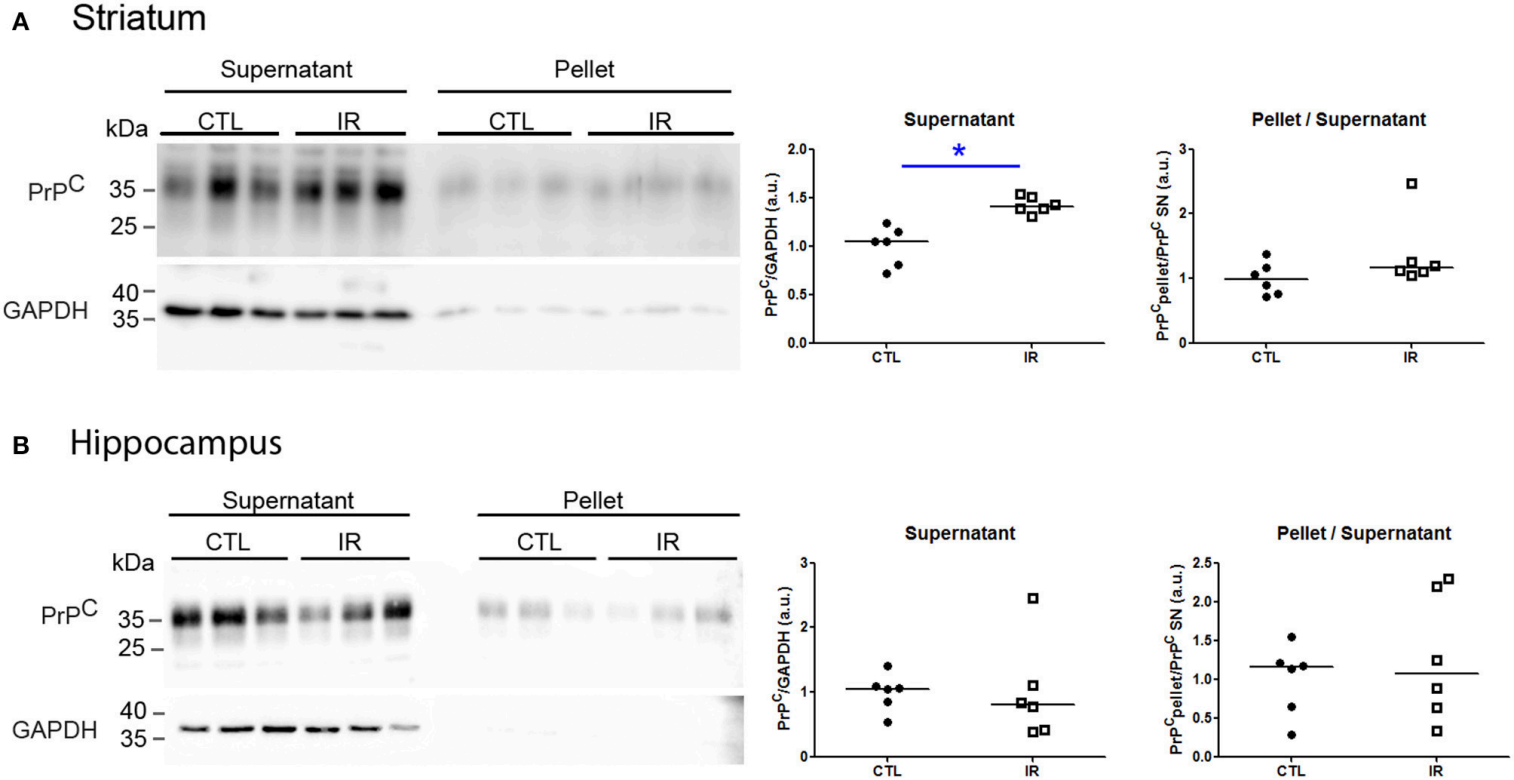
**Analysis of PrP^C^ solubility**. Soluble (supernatant) and insoluble (pellet) fractions were separated by ultracentrifugation and PrP^C^ and GAPDH were detected by Western blotting. Left panels show representative images of each region. To estimate relative amount of PrP^C^ in supernatant, PrP^C^ band intensity was divided by respective GAPDH signals. The ratio of insoluble PrP^C^ was calculated by dividing the intensity of pellet signals by respective supernatant signals. All results were normalized by mean of CTL group. Each dot represents result of an animal and horizontal line represents median of the group. **(A)** Striatum (*n* = 6 per group) (B) Hippocampus (*n* = 6 per group). ^*^p < 0.05.

Protein aggregation is a concentration-dependent process. As dietary iron restriction increased PrP^C^ levels in the striatum, we verified whether this increase affected its solubility. Insoluble protein oligomers (pellet) were recovered by ultracentrifugation, and PrP^C^ of each fraction was detected by Western blot. To improve the signal detection in pellet fractions, 20-fold higher equivalent amounts of pellet were loaded into gel compared to supernatants. The striatum of IR group presented higher amount of PrP^C^ in the supernatant compared to CTL group, but both groups presented similar scarce amount of PrP^C^ in pellet fraction (Figure 8A), indicating that enhanced expression level of PrP^C^ did not culminate into aggregation.

Hippocampus is one of the brain regions that express highest level of PrP^C^ (Tanji et al., 1995; Salès et al., 2002). Moreover, PrP^C^ is known to play antioxidant roles with the ability to directly react with ROS, producing insoluble oligomers (Bertuchi et al., 2012; da Luz et al., 2015). Since we observed higher levels of lipid peroxidation in the hippocampus of IR group, we verified whether dietary iron restriction was also capable of changing the PrP^C^ solubility. As shown in Figure 8B, both groups presented similar amount of PrP^C^ in pellet and supernatant. Nevertheless, relatively weak GAPDH signal in pellets reinforces that PrP^C^ is more prone to aggregate than GAPDH.

Altogether, these findings demonstrate that chronic insufficiency of dietary iron supply evokes distinct responses in striatum, prefrontal cortex and hippocampus with respect to iron and dopamine metabolism and proteostasis.

## Discussion

Conformational changes in proteins and deposition of their aggregates are common hallmarks of several neurodegenerative diseases. Thus, identification of conditions that lead to protein aggregation is important not only for elucidation of mechanism of pathogenesis, but also to establish diverse strategies of prevention and treatment. Many studies have demonstrated a close relationship between iron and dopamine metabolism and neurodegenerative diseases (Belaidi and Bush, 2015; Hare and Double, 2016). In this study, we aimed to disturb dopamine metabolism by restricting dietary iron and investigate how altered iron and dopamine metabolism affect proteostasis of PrP^C^ and α-synuclein.

Previous studies have demonstrated that 30 or less days of dietary iron restriction were sufficient to mimic iron deficiency in animal models with reduced iron and ferritin levels in liver and serum (Youdim et al., 1989; Kamei et al., 2013). Indeed, we observed reduced iron and ferritin levels in liver of IR group as expected. However, we did not observe alterations in serum iron levels although serum ferritin level was reduced. This lack of correlation between total iron pool and ferritin levels in serum can be explained by abundant presence of other iron binding proteins such as transferrin, albumin and free hemoglobin. Indeed, red blood cells are considered one of largest iron pools. Considering that half-life time of red blood cells or hemoglobin of mice is more than 30 days and most of iron derived from red blood cells are recycled and not excreted (Vanputten, 1958; De Domenico et al., 2008; Muñoz et al., 2009), it is reasonable to expect that 30 days of nutritional iron restriction do not significantly deplete the iron pool in blood. In addition, lack of a significant increase of transferrin receptor in all tissues evaluated in this study also indicates that our experimental condition was not sufficient to fully exhaust iron stores (Skikne et al., 1990; Cook, 2005). In younger animals, shorter period of iron restriction can robustly increase the expression of transferrin receptor (Moos et al., 1998), suggesting that age is an important factor that influence the responses to dietary iron restriction.

In addition to partial depletion of peripheral iron pools, we also observed reduced ferritin levels in hippocampus, indicating that brain iron metabolism was also affected by our experimental protocol. Concomitant with ferritin reduction, higher degree of lipid peroxidation was observed in the hippocampus of iron restricted animals. Iron deficiency is known to reduce cytochrome C activity and energy metabolism, leading to increased production of reactive oxygen species (de Deungria et al., 2000; Carlson et al., 2009; Srinivasan and Avadhani, 2012). Moreover, iron deficiency can reduce mTOR activity in hippocampus via REDD1, a signaling molecule that can be activated by reactive oxygen species (Ohyashiki et al., 2009; Waibel et al., 2009). Thus, our observations appear to corroborate these previous findings that show increased oxidative stress in hippocampus under iron deficient condition.

Intriguingly, ferritin level was increased in the striatum of iron-deprived animals. This result was somewhat similar to the results reported in previous study, which showed that the treatment of younger rats with iron deficient diet reduced the iron level in hippocampus, but not in striatum (Erikson et al., 1997). Iron distribution in the normal brain is known to be heterogeneous (Hill and Switzer, 1984; Rouault, 2013), and striatum is one of the regions that have higher iron levels in the adult brain and is one of the sites where iron accumulates during normal aging (Martin et al., 1998). Altogether, these findings suggest that each brain region has distinct iron requirements, and under iron deficient condition, each region might develop distinct capacity of iron storage by altering the ferritin expression level. Nonetheless, abnormal ferritin accumulation in the striatum has been referred to as an early event in neurological motor dysfunction such as Parkinsonism and Huntington disease (Vidal et al., 2004; Oakley et al., 2007; Simmons et al., 2007). Although underlying mechanisms needs to be further investigated, our data show that chronic nutritional iron deficiency can trigger molecular alterations observed in early stage of neurological disorders that involve degeneration of dopaminergic neurons.

Similar to ferritin levels that varied in a region-specific manner, dopamine metabolism of each region also generated distinct outcomes in animals subjected to dietary iron restriction. In hippocampus, which receives relatively small portion of dopaminergic innervation from VTA, iron restriction did not cause any significant alterations. In prefrontal cortex, which receives innervation from same region, dopamine or DOPAC levels were also not altered in IR group. However, HVA levels were increased probably due to the increased MAO-A activity. Previous studies have demonstrated that iron chelators can reduce the MAO activity, suggesting that iron bioavailability can affect the MAO activity (Zheng et al., 2005; Gal et al., 2006). The underlying mechanism of induction of MAO-A activity in IR group is still unclear, but these results can help to explain the symptoms of depression reported in iron-deficient population (Corwin et al., 2003), since increased MAO-A activity has been linked to mood disorders (Meyer et al., 2009; Sacher et al., 2015; Kolla et al., 2016).

Reduced levels of dopamine and/or its metabolites in striatum of IR group were rather expected based on previous observations (Unger et al., 2014; Matak et al., 2016). It is tempting to speculate that this reduction occurred due to the compromised dopamine synthesis, since iron is a cofactor for tyrosine hydroxylase, an enzyme that catalyzes limiting step of dopamine synthesis. On the other hand, iron deficiency is known to evoke abnormal signaling via D2 receptor (Youdim et al., 1989; Erikson et al., 2001; Matak et al., 2016). Activation of D2 auto receptors regulates dopamine release and TH activity (Ford, 2014). Thus, it is possible that abnormal D2 receptor signaling contributed to reduced levels of dopamine metabolites in striatum of iron deprived animals.

Since extensive studies have demonstrated the involvement of PrP^C^ and α-synuclein in dopamine and iron metabolism, we evaluated how these divergent outcomes of dietary iron restriction in each region affected the proteostasis of both proteins. Regarding the expression levels, only PrP^C^ of the striatum was significantly increased after iron restriction. Previous studies have demonstrated that high concentration of dopamine can promote oxidation and oligomerization of PrP^C^ (Shiraishi et al., 2005; da Luz et al., 2015). PrP^C^ can also regulate dopamine metabolism by suppressing the TH expression (Beckman et al., 2015; da Luz et al., 2016). However, in this study, we observed that iron restriction reduced the levels of dopamine metabolites in the striatum, without affecting TH levels or PrP^C^ solubility. Thus, increased PrP^C^ levels observed in this region might not be related with dopamine metabolism. On the other hand, PrP^C^ is known to play roles in iron uptake by acting as ferrireductase, and its expression appears to correlate with ferritin expression (Singh et al., 2013; Haldar et al., 2015; Tripathi et al., 2015). Thus, the increase of PrP^C^ in striatum of IR group is more likely related to iron recruitment into the striatum leading to the increased ferritin level. Moreover, the regulation of ferritin and PrP^C^ expression might play important roles for intracellular iron buffering capacity since dietary iron restriction did not alter expression levels of α-synuclein in any brain regions, despite previous report showing reduced translation of α-synuclein upon iron chelation (Febbraro et al., 2012). These results also reinforce specificity of the iron restriction effects on proteins of distinct subcellular comportments.

Altogether, our results demonstrate that insufficient dietary iron supply can establish molecular features that resemble several neurological disorders, such as abnormal ferritin accumulation in striatum observed in parkinsonian syndromes and Huntington disease (Vidal et al., 2004; Simmons et al., 2007), increased MAO-A activity in prefrontal cortex which is associated with depression (Meyer et al., 2009) and increased degree of lipid peroxidation in hippocampus that is related with most of conformational diseases (Sultana et al., 2013). By showing these molecular alterations, this study emphasizes the importance of adequate iron supplementation for brain health and prevention of neurological diseases. Moreover, these brain region-specific responses can be useful information to better understand the diversity of the disorders that can be generated by altered iron metabolism.

## Author contributions

JP conducted all experiments. Md assisted in animal handling and western blotting. HA and SG assisted in animal handling and sample collection. VM helped with the study design and interpretation of data, and revised the manuscript. KL conceived and supervised the study, and wrote the manuscript. All authors contributed to final version of the manuscript.

## Funding

This study was supported by grants from FAPESP (Fundação de Amparo à Pesquisa do Estado de São Paulo: 2016/04297-6), CNPq (Conselho Nacional de Desenvolvimento Científico e Tecnológico: 467566/2014-3), CAPES (Coordenação de Aperfeiçoamento de pessoal de Nível Superior) and EMU (programa de equipamentos multiusuários).

### Conflict of interest statement

The authors declare that the research was conducted in the absence of any commercial or financial relationships that could be construed as a potential conflict of interest.

## Abbreviations

PrP^C^: Cellular Prion Protein
IR: iron restricted diet
CTL: control diet
TH: tyrosine hydroxylase
DAT: dopamine transporter
DOPAC, 3: 4-Dihydroxyphenylacetic acid
HVA: homovanillic acid
MAO: Monoamine oxidase.
